# News and Events of the Week

**Published:** 1910-04-16

**Authors:** 


### News and Events of the Week.

Sir Henry Burdett's address at the Home Hospitals Association on "The Future of the Hospitals," which was reported in our issue of March 26 last, has been reprinted in pamphlet form, and is being sold at 2*d.* a copy. All interested in the future of medical relief and its provision for all classes on a provident basis should procure a copy of this pamphlet, wherein will be found an outline of a scheme of co-operation by means of insurance between patients, the hospitals, and the State. The publishers are the Scientific Press, 28 and 29 Southampton Street, Strand, W.C.

The annual general meeting of the London and Counties Medical Protection Society, Ltd., will be held at 31 Craven Street, Strand, London, on Wednesday, April 27, 1910, at 4 p.m., to receive and adopt the annual report and balance-sheet; to elect the president, vice-presidents, council, officers, and auditors; and to transact other business.

The Committee of the Athenæum Club on Tuesday elected several gentlemen under the provisions of Rule 2 of the club, which empowers the annual election by the Committee of nine persons "of distinguished eminence in science, literature, the arts, or for public services," among whom was Sir James Reid, Bart., K.C.B., G.C.V.O., Physician in Ordinary to the King and to the Prince of Wales.

The display which the London County Council has arranged to send to the Japan-British Exhibition will occupy an area of 4,000 square feet, and will include models of Battersea Park, cottages on one of the Council's housing estates, tenement fittings, Bruce House showing the cubicles, and the Inebriates' Infirmary. With these will be exhibited a representation of an old-type common lodging-house. There is to be an exhibit illustrating the educational work of the Council. Models will also be shown of Holborn and Kingsway, the Barking sewage scheme, and the underground railways.

The National Society of Day Nurseries has a very interesting exhibit at the Ideal Home Exhibition which opened at Olympia yesterday. All those interested in crèche work will have an opportunity of seeing what is being done to provide suitable accommodation for babies whose mothers have to go out to work daily. The Society is exhibiting a model for the guidance not only of those about to establish a crèche, but also of those who desire in existing day nurseries to reach the standard of efficiency requisite before affiliation with the Society can be obtained.

A "Civilian Doctor," writing to a London daily newspaper in connection with the recent hydrophobia case, gives a striking example of Army red tape. A case was recently brought to my notice where an officer had been sent home from India, after a very severe attack of enteric, on the recommendation of a medical board. The unfortunate doctor who had attended the case and recommended that the officer should be sent home was informed that if a similar case occurred he would be liable for the cost incurred. The Government of India considered that the patient should have been sent to the hills in India instead of home—an absurdity, as the officer was actually serving in a hill station during his illness.

Mr. Ernest Herbert Cobb, M.R.C.S., L.R.C.P., of Belmont, Stevenage, Herts., who died on February 15, in his fiftieth year, has left estate valued at £55,660 gross and £53,936 net.

A lecture will be delivered at the Royal Sanitary Institute, 90 Buckingham Palace Road, S.W., by the Hon. Sir John A. Cockburn, K.C.M.G., M.D., on "The Public Health Aspect of Food Supply in the Colonies," on Wednesday, April 20, at 5.15 p.m. The President, his Grace the Duke of Northumberland, K.G., P.C., F.R.S., will take the chair.

All the medical libraries in Berlin are about to be organised into the Association of Medical Libraries, of which Professor E. A. Ewald will be president. The Association will probably include the libraries of the Berlin Medical, the German Surgical, the Dermatological, Laryngological, Otological, and Röntgenological Societies, besides those of the Imperial Committee for the Study of Cancer, and the Medical Department of the Empress Frederick Foundation.

The "Administration of Anæsthetics" Committee appointed by the Home Secretary in 1908 to inquire into the law relating to coroners' inquests has issued its report on deaths resulting from anæsthetics. It states that there is an increasing number of deaths under anæsthetics, and urges that their administration should be regulated by law. The committee recommends that the administration of anæsthetics the effect of which is prolonged should be confined to qualified medical men.

Under the patronage of Lady Cromer, Lady Henry Somerset, Sir Thomas Barlow, and others, an exhibition for the prevention of tuberculosis, organised by the N.A.P.C., is now open in St. Pancras till April 23. Arrangements have been made for the use of the Maurice Hall, Working Men's College, Crowndale Road, where the exhibition will be open from noon to 10 p.m. daily. Curators will be in attendance to explain the exhibits, and popular lectures will be given by some of the principal authorities each evening at 8.30 p.m. Admission free.

Medical Men and the Peace Movement.—In 1905, when the horrors of the Russian-Japanese war were attracting universal attention, an International Committee of medical men to contribute to the suppression of the war was formed at Paris, mainly through the instrumentality of Dr. J. Rivière. This Committee has since gradually grown in numbers, and has broadened its scope, so that it is to-day an international medical peace committee, which every medical man, who is in favour of peace and believes that such efforts as are being made by societies which have in view the cultivation of international amity and goodwill are conducive towards the attainment of peace, should join. On the executive committee of this Association are such well-known physicians and surgeons as Adamkiewicz, d'Arsonval, Bacelli, Bouchard, Bourget, Crichton-Browne, Ramon y Cajal, Lucas Championniere, Erb, Fournier, Hayem, Landouzy, Reclus, Reverdin, Roux, Sahli, Weichselbaum, and Rosenthal. The Association has decided to hold its first annual Congress at Paris in June, 1911. The General Secretary of the movement is Dr. J. Mazery, 25 Rue des Mathurins, Paris, to whom all applications should be addressed.

